# Otologic Manifestations of Temporomandibular Disorders

**DOI:** 10.3390/diagnostics16121757

**Published:** 2026-06-07

**Authors:** Fatemeh Ebrahimi, Ali Akbar, Vivian Jin, Vivian F. Kaul, Craig B. Pearl

**Affiliations:** 1 Katz Department of Oral and Maxillofacial Surgery, School of Dentistry, The University of Texas Health Science Center at Houston, Houston, TX 77054, USA; fatemeh.ebrahimi@uth.tmc.edu (F.E.);; 2 Department of Surgical Sciences, College of Dentistry, Health Sciences Center, Kuwait University, Kuwait City 13060, Kuwait; 3 Department of Otolaryngology Head and Neck Surgery, UTHealth Houston McGovern Medical School, Houston, TX 77030, USA; vivian.jin@uth.tmc.edu (V.J.); vivian.f.kaul@uth.tmc.edu (V.F.K.); 4 Department of Oral and Maxillofacial Surgery, Memorial Hermann Hospital, Texas Medical Center, Houston, TX 77030, USA

**Keywords:** temporomandibular joint disorder, ear disease, otologic symptoms, hearing loss, aural vertigo, tinnitus, otalgia

## Abstract

**Background/Objectives**: Temporomandibular disorder (TMD) affects a third of the adult population and has been associated with otologic symptoms. These symptoms are frequently misattributed to primary otologic diseases, leading to delays in diagnosis and treatment. This review aims to summarize the reported prevalence, proposed pathophysiologic mechanisms, and management strategies of otologic manifestations in patients with TMD. **Methods**: A literature review was conducted using the MeSH terms “temporomandibular joint disease” and “otologic symptoms.” Five additional searches were performed using “temporomandibular disease/dysfunction” combined with each of the five most common otologic symptoms. Meta-analyses, randomized controlled trials, reviews, and systematic reviews were prioritized, with preference for studies published within the last 10 years. Inclusion criteria focused on human studies addressing the etiology, clinical presentation, and management of otologic symptoms in TMD populations. **Results**: The literature supports an association between TMD and otologic symptoms in the absence of primary ear disease. The most commonly described symptoms were aural fullness, otalgia, tinnitus, vertigo, and hearing loss. Conservative approaches, including occlusal splints, physical therapy, behavioral modification, and pharmacologic therapy, demonstrated partial or complete symptom resolution after management of underlying TMD. **Conclusions**: The literature demonstrates a consistent association between otologic symptoms and TMD, although the underlying mechanisms remain incompletely understood. While conservative TMD management may improve symptoms, exact mechanisms remain unproven. Clinicians should consider TMD in the differential diagnosis when patients present with unexplained otologic complaints. Further research is necessary to establish causality, confirm the efficacy of management protocols, and improve diagnostic accuracy in this overlapping domain.

## 1. Introduction

Temporomandibular disorder is a broad term used to describe any condition presenting with chronic pain or dysfunction of the temporomandibular joint (TMJ) and/or associated muscles, including but not limited to the muscles of mastication as well as the cervical muscles and strap muscles. TMD is estimated to affect as much as a third of the adult population, with a predilection for young and middle-aged females [1]. Given its high prevalence, the financial implications are also significant. In 1999 alone, treatment for TMD-related conditions in the United States was estimated to cost approximately USD 21.4 billion [2].

Temporomandibular disorders are most commonly associated with parafunctional habits such as clenching, grinding, or trauma [3]. Less commonly, pathological conditions, including cysts and tumors involving the temporomandibular joint, may present TMD-like symptoms [2]. Benign neoplasms of the bones are rare, accounting for only 0.2% of all human tumors. Those originating in the TMJ are extremely rare [4]. The clinical presentation of patients with benign tumors and cysts of the temporomandibular joint often may initially present as TMD that develops because of the benign pathology and its effect on surrounding tissues. These patients will frequently present with symptoms such as pain, limited mouth opening, deviation of mandibular movements, crepitus, and malocclusion [5,6]. TMJ disorders are perhaps one of the most frequently misdiagnosed or undiagnosed problems that afflict as much as 33% of the population. At any given time, between 7% and 12% of these patients seek treatment for TMD-associated symptoms [7].

Due to the similar early presentation of benign pathology of the TMJ, when patients present to an Oral and Maxillofacial Surgeon, they often have signs and symptoms that would lead to a provisional diagnosis of TMD with possible disc displacement with or without reduction or varying degrees of degenerative joint disease. The most common of these signs and symptoms is pain in the preauricular area with mouth opening, limited mouth opening, joint noises such as clicking, grating, or crepitus, and a history of inability to open the jaw, or less frequently, close the jaw after opening wide. These specific TMJ symptoms are frequently associated with tenderness over the muscles of mastication. Patients describe a generalized pain over one or both sides of the face in the region of the masseter muscles, headaches, or a tension-type pain in the region of the temporalis muscles, with pain referring to behind the eyes or ears. Similarly, patients with benign neoplasms and cysts will frequently present with symptoms that are very similar to TMD. The pathogenesis of this is thought to be associated with the expansion of these lesions into the surrounding tissues [8]. Other causes of temporomandibular disease include inflammatory disorders or arthritis, adhesions from loss of lubrication between structures, disc dislocation, synovitis or capsulitis, or growth disorders causing hyperplasia.

Temporomandibular disorders often mimics other conditions, and the pathogenesis is multifactorial, leading to misdiagnosis with resultant delays in treatment. The unique predilection of TMD to present with a wide variety of often unrelated symptoms has led to extensive research aimed at better understanding the complex etiology and the unique sequelae of these symptoms [1]. A significant number of patients that are referred to an oral and maxillofacial surgeon are referred by an otolaryngologist. These patients typically describe one or more of the following symptoms: blocked or a feeling of fullness in the ears, pain radiating from the ear, tinnitus, vertigo, and, less frequently, hearing loss [9]. Otologic symptoms of TMD have been reported throughout the literature, but the connection is often initially unsuspected, which can result in further delays in diagnosis. In the 1970s, the term Otomandibular Syndrome was coined by a practicing otolaryngologist, Dr. Harold Arlen, to describe a group of patients with otalgia, aural fullness, hearing loss, tinnitus, and vertigo without any ear, nose, and throat (ENT)-related causes on examination [10].

In the ENT setting to work up otalgia, typically otoscopy, formal audiologic testing, and imaging are performed for initial workup. In the absence of findings from this workup and in conjunction with clinical symptoms of bruxism or reproduced condylar tenderness, the patient may then be referred to an Oral and Maxillofacial Surgeon (OMS) for further evaluation for TMD. A systematic review and meta-analysis from the Journal of Clinical Oral Investigations stated that ear fullness (74.8%), otalgia (55.1%), tinnitus (52.1%), vertigo (40.8%), and hearing loss (38.9%) are the most common otologic signs and symptoms reported in patients with temporomandibular disorders [2]. While otologic symptoms associated with temporomandibular disorders are well-described in the English literature, the exact mechanism, as well as the ideal management for these symptoms remains unclear [11,12]. Through a narrative review of the literature, this paper seeks to summarize the reported prevalence, proposed pathophysiologic mechanisms, and management of common otologic manifestations associated with TMD.

## 2. Methods

Most research on otologic symptoms associated with TMD is published in Otolaryngology journals [12]. There is a paucity of publications on otologic symptoms associated with temporomandibular disorders in the oral and maxillofacial literature. The exact mechanisms, pathophysiology, and management are not well described in the English literature.

To investigate the correlation between otologic symptoms and temporomandibular disorders, a comprehensive literature search was conducted using the PubMed database through May 2025. The literature search was conducted using combinations of MeSH and free-text keyword terms related to temporomandibular disorders and otologic symptoms. The primary search terms included “temporomandibular disorders”, “temporomandibular dysfunction”, “otologic symptoms”, “tinnitus”, “otalgia”, “aural fullness”, “vertigo”, and “hearing loss.” Boolean operators (AND/OR) were used to combine search terms, and additional targeted searches were performed for each of the five most common symptoms outlined previously (ear fullness, otalgia, tinnitus, vertigo, and hearing loss). Additional searches were conducted using the following combinations: “temporomandibular dysfunction and otologic symptoms”, “temporomandibular dysfunction AND/OR ear fullness”, “temporomandibular dysfunction AND/OR otalgia”, “temporomandibular dysfunction AND/OR tinnitus”, “temporomandibular dysfunction AND/OR vertigo”, and “temporomandibular dysfunction AND/OR hearing loss”.

Search results were limited to studies published in English involving human subjects. Eligible publication types included observational studies, randomized controlled trials, systematic reviews, meta-analyses, narrative reviews, case series, and case reports. Animal studies, non-English publications, and studies not evaluating the relationship between TMD and otologic manifestations were excluded.

**Figure 1:**
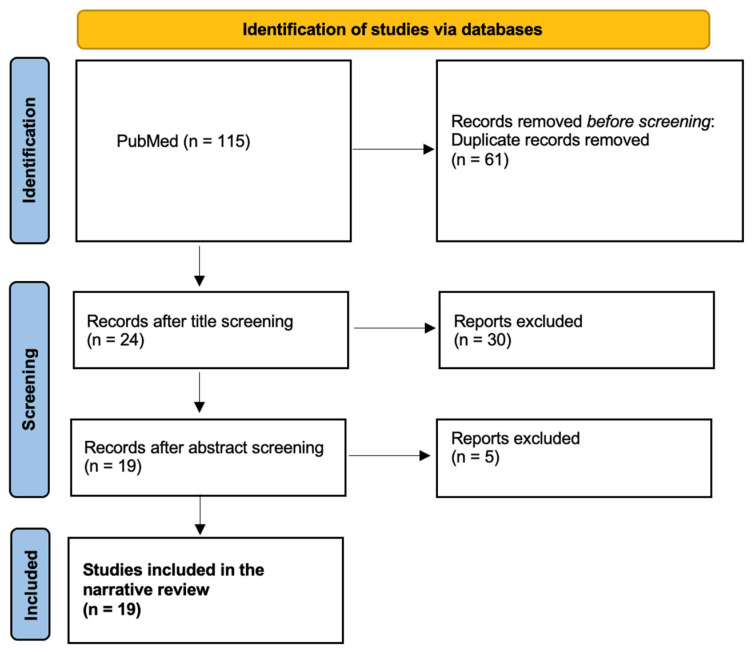
PRISMA diagram representing the screening strategy and selection process for eligible research articles.

A total of 115 records were identified through the initial search. Following removal of duplicate records (*n* = 61), 54 articles underwent title screening. Thirty studies were excluded because they were unrelated to the objectives of the review or did not evaluate otologic symptoms associated with TMD. Twenty-four articles proceeded to abstract screening, during which five additional studies were excluded because they lacked relevant clinical outcomes or discussion of TMD-associated otologic manifestations. Nineteen studies met the eligibility criteria and were included in the final narrative review. The study selection process is summarized in Figure 1 using a PRISMA-style flow diagram documenting records identified, screened, excluded, and included.

Studies were included if they reported the prevalence, pathophysiology, diagnosis, clinical presentation, or management of otologic symptoms associated with TMD. Given the narrative nature of this review, studies employing different diagnostic criteria for TMD and varying methods of otologic symptom assessment were considered eligible when they contributed relevant evidence to the understanding of TMD-related otologic manifestations. Diagnostic approaches and symptom definitions were reviewed and interpreted within the context of each individual study. The pertinent findings from this literature review are discussed below to provide context to the variety of causes that may be the source of these symptoms as well as the proposed pathophysiologic mechanism connecting them to TMD.

## 3. Results

The included publications consisted of systematic reviews, randomized clinical trials, cohort studies, cross-sectional studies, and case reports evaluating both the prevalence of otologic symptoms in TMD populations and the effect of TMD-directed therapies on symptom improvement. While study design and patient populations varied, the overall body of literature consistently reported an association between temporomandibular disorders and ear-related symptoms in the absence of identifiable primary otologic disease.

The most commonly described complaints include aural fullness, otalgia, tinnitus, vertigo, and hearing loss. These symptoms are frequently encountered in both dental and otolaryngology settings and are often part of the initial presentation that leads patients to seek evaluation. Many investigations report that patients experience more than one of these symptoms simultaneously, suggesting that otologic manifestations may represent a broader clinical pattern rather than isolated findings.

Studies evaluating management strategies frequently reported improvement in otologic symptoms following treatment of TMD. Conservative therapies such as occlusal splints, physical therapy, behavioral modification, and pharmacologic management were the most commonly described approaches. Although treatment protocols vary, many studies described partial or complete resolution of symptoms following management of the underlying TMD. The principal findings of the included studies are summarized in Table 1.

## 4. Discussion

### 4.1. Aural Fullness

#### 4.1.1. Overview and Etiology

Aural fullness is the most reported otologic symptom associated with TMD [2,13]. Many proposed etiologies exist that may result in the patient reporting a sensation of pressure, congestion, or blockage in the ears, including eustachian tube dysfunction (ETD), neuromuscular dysfunction, and other related anatomical or functional abnormalities [13]. The most common of these etiologies is ETD, which is failure of the eustachian tube to achieve pressure equalization between the middle ear and external environment [13,31]. The etiologies of ETD are multifactorial and may be due to infections, allergies, developmental anomalies, or anatomical obstructions [32]. Chronic eustachian tube dysfunction may lead to prolonged tympanic membrane retraction. This can be visualized on otoscopy and is often represented by abnormal tympanograms on formal audiologic testing, suggesting negative pressure in the middle ear.

In otolaryngology practice, the primary treatment approach for ETD in adult patients with identifiable otologic pathology involves intranasal corticosteroids and antihistamine nasal sprays to decongest and reduce inflammation around the eustachian tube opening. In cases where medical management is unsuccessful, surgical interventions such as myringotomy, with or without ventilation tube placement, or balloon dilation of the eustachian tube may be considered to relieve chronic negative pressure [32]. It is important to note that aural fullness does not always equate to Type C tympanograms, which are indicative of negative pressure in the middle ear. In many cases, aural fullness is a patient-specific symptom rather than an obvious sign or finding that can be followed over time. For patients with a normal otologic exam, alternative causes, such as temporomandibular joint disorders, should be considered and further evaluated based on commonly associated symptoms, including clicking or crepitus, limited mandibular mobility or deviation during opening, masticatory discomfort, and others [13,14].

From the dental literature standpoint, a review in the Journal of the American Dental Association (JADA) proposed that neuromuscular dysfunction, particularly a hyperactive medial pterygoid and tensor tympani muscles, may contribute to aural fullness in patients with temporomandibular pain and an otherwise normal otologic exam [13,31]. The temporomandibular joint and the eustachian tube share a common embryologic origin from Meckel’s cartilage, which is involved in developing the jaw and surrounding structures. Accordingly, the mandibular branch of the trigeminal nerve (CN V) innervates the muscles of mastication, as well as the tensor tympani and tensor veli palatini muscles. The tensor tympani muscle influences the position and contraction of the tympanic membrane, while the tensor veli palatini muscle regulates the tension of the soft palate and opening of the eustachian tube. The torus tubarius, a mucosal fold located in the nasopharynx, is primarily innervated by the glossopharyngeal nerve and additional branches via the pharyngeal plexus. Specifically, the anterior wall of the torus tubarius receives innervation from the nerve to the tensor veli palatini. The torus tubarius provides a structural framework for the cartilaginous portion of the eustachian tube, helping to maintain its shape and position. Chronic bruxism or teeth grinding can lead to inflammatory changes within the jaw muscles, as well as surrounding structures and nerves [33]. Changes in the nerve to tensor veli palatini may impact not only the function of the tensor veli palatini muscle itself but also the anterior fold of the torus tubarius, thereby disrupting the structure, function, and patency of the eustachian tube. These disruptions can impair pressure regulation within the middle ear.

In a 2017 prospective cohort study, 112 patients with a presumed non-otolaryngological origin of aural fullness were evaluated. These patients exhibited signs and symptoms of TMD and were treated according to disease categories and therapeutic responsiveness. Among the study participants, 90.2% reported either complete resolution or significant improvement of aural fullness following TMD treatment. TMD therapies, especially physical therapy, proved highly effective in patients with muscle-related TMD (Table 2). However, treatments were often ineffective in those with structural temporomandibular joint changes. This study established a correlation between aural fullness and TMD in the patient cohort [14].

One case report describes a patient with debilitating unilateral ear fullness and temporary hearing loss that was not linked to any middle ear pathology upon evaluation by an otolaryngologist [15]. After placement of ventilation tubes in the tympanic membrane was unsuccessful in providing long-term relief, the patient was referred to a dental clinic for further evaluation. Comprehensive clinical evaluation revealed a limited range of motion and crepitus associated with the left temporomandibular joint. Magnetic resonance imaging revealed moderate bilateral degenerative changes with associated bilateral disc displacement without reduction. The final diagnosis in this case was localized TMJ osteoarthritis with associated masticatory myofascial pain syndrome, primarily involving the left masseter and tensor veli palatini muscles. Treatment for this patient was carried out over three months. It was a multimodal approach that included at-home physical therapy, utilization of a full-arch stabilization splint, trigger point injection of the left superficial masseter with 0.5 milliliters of plain 2% lidocaine, and a 0.5 milliliter injection of triamcinolone acetonide mixed with 1.5 milliliters of 2% lidocaine into the left superior temporomandibular joint space. At the end of this three-month treatment, the patient reported a 90% improvement in her otologic symptoms, including not only aural fullness, but her temporary hearing loss. This case highlights the potential clinical manifestations of aural fullness, the importance of identifying its etiology, and the effectiveness of tailored management strategies.

TMD is often overlooked as the causative agent in a patient with complaints of aural fullness. In one study in JAMA Otolaryngology, symptoms of aural fullness were present in 74% of TMD patients referred to an ENT clinic [34]. Another study demonstrated that 46.07% of patients experiencing aural fullness had abnormal audiogram results, indicating that aural fullness may occur as both an objective and subjective symptom in nearly equal proportions [35]. Orofacial myofunctional therapy has been shown to successfully resolve aural fullness symptoms in various case reports and limited case series. However, larger-scale studies are necessary to establish their efficacy and determine its suitability as a standard treatment for TMD-associated aural fullness [14,15,16].

#### 4.1.2. Management of TMD-Related Aural Fullness

While TMD treatment approaches vary, conservative therapies are commonly used to address TMD-related symptoms. In our experience, primary management typically includes occlusal splints, pharmacological interventions (e.g., non-steroidal anti-inflammatory drugs, muscle relaxants), physical therapy, and stress management techniques. In more severe cases, intra-articular injections, arthrocentesis, arthroscopy, or surgery may be considered.

In a 2016 systematic review of the effect of conservative TMD treatment on otologic symptoms, the authors concluded insufficient evidence in favor of or against conservative therapies for TMD on changes in otologic signs and symptoms [17]. Supporting these studies in a more recent prospective study, Naderi et al. (2023) [18] evaluated TMD treatments in 40 patients, with a focus on otologic symptoms. The treatment protocol included a soft diet, stretching exercises, thermal compresses, elimination of parafunctional habits, physiotherapy, occlusal splints for patients with parafunctional habits, and case-specific medications. At 2-week and 2-month follow-ups, patients self-reported improvements in otologic symptoms using the Visual Analog Scale (VAS). Of the 40 participants, 12 had ear fullness as a primary complaint. After 2 weeks, 57.8% reported improvement, with 50% noting significant improvement. At 2 months, 82.8% reported improvement, with 46.3% reporting significant improvement of aural fullness [18]. Given the inconclusive nature of current evidence, further research is needed to optimize treatment protocols and evaluate the effectiveness of interventions for managing these cases.

### 4.2. Otalgia

#### 4.2.1. Overview and Etiology

**Figure 2:**
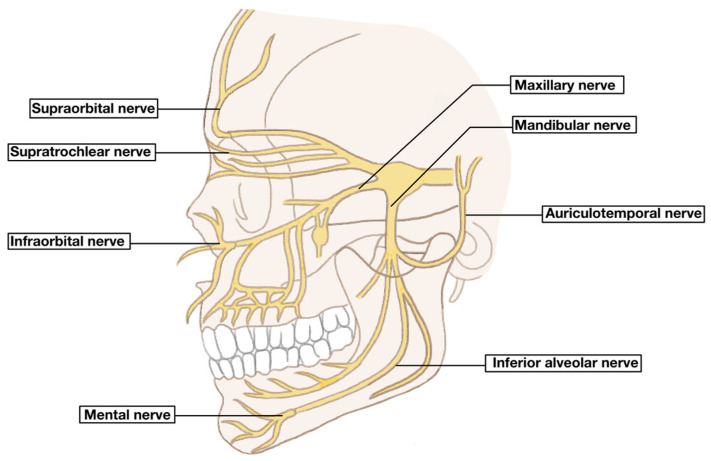
Sensory innervation of the craniofacial region. The auriculotemporal nerve (mandibular division of the trigeminal nerve, CN V3) is highlighted as a potential neuroanatomic pathway linking temporomandibular disorders to referred otalgia.

Pain in the preauricular region occurs when the area located immediately anterior to the ear presents with tenderness upon palpation or manipulation of the jaw at the periauricular region. Palpation of the lateral pterygoid muscle and the stylomandibular ligament, both located intraorally along the inner cheek, can occasionally elicit referred pain in the preauricular region. Patients may also report exacerbation of symptoms when consuming tough or crunchy foods, or a clinical history suggestive of bruxism. Evaluating otalgia requires an understanding of both the ear’s sensory innervation and the overlapping neural pathways with areas in the head and neck [36]. Proximity to cranial nerves V, VII, IX, and X along with the C2 and C3 branches of the cervical plexus can cause distant referred pain to this region that can further complicate management [37]. Structures and mechanisms proposed to contribute to otalgia in patients with temporomandibular disorders are summarized in Table 3. Of particular importance to this discussion is the auriculotemporal branch of CN V that innervates the temporomandibular joint creating a clear connection between TMD and otalgia [19]. In relation to the ear, parasympathetic innervation originates from the inferior salivatory nucleus, where cranial nerve IX (Jacobsen’s nerve) enters the middle ear through the tympanic canaliculus. This pathway joins the lesser superficial petrosal nerve (LSPN), which passes through the foramen ovale to reach the otic ganglion. From there, sensory fibers continue via the auriculotemporal nerve. Given the anatomical proximity of the temporomandibular joint to the external auditory canal, reactive inflammation or irritation of the auriculotemporal nerve has been proposed as a potential mechanism for referred pain to the ear, clinically presenting as otalgia. However, this relationship remains largely inferential and has not been definitively established. In addition to nerve irritation, various mechanisms involving the masticatory muscles, which are in direct functional and anatomical proximity to the ear, have been proposed to explain otalgia in the TMD population (Figure 2) [15].

When the preauricular area is painful, the differential diagnoses must first determine whether the source is primary or otogenic otalgia, originating within the ear, or referred otalgia, originating from outside the ear (Table 4) [19]. Common causes of primary otalgia include cotton swab trauma, foreign body in the ear, acute otitis media (AOM), otitis externa, eustachian tube dysfunction, cholesteatomas, and neoplasms of the ear [37].

Secondary otalgia, often presenting with periauricular pain, is frequently attributed to conditions such as maxillary sinusitis, trigeminal neuralgia, parotid gland disease, temporomandibular joint synovitis and, notably, temporomandibular musculoskeletal dysfunction as cited in the literature [19,43]. Masticatory muscle hyperactivity may contribute to referred ear pain by increasing the tonicity of the tensor veli palatini and tensor tympani muscles [44,45]. Although primary etiologies are the most common causes of otalgia, temporomandibular dysfunction is the most prevalent source of secondary otalgia, particularly among women aged 20 to 40 years, and may account for as much as 50% of overall cases of otalgia [46,47]. Less common causes of secondary otalgia include cerebellopontine angle tumors, geniculate neuralgia, tonsillitis or pharyngeal lesions referring pain via Jacobsen’s nerve, laryngeal lesions affecting Arnold’s nerve, and cervical spine degeneration involving the great auricular nerve [48]. The frequency of specific diagnoses may vary depending on whether the patient is first assessed by an otologist, neurologist, or another physician, underscoring the need for a detailed physical examination of symptoms to prevent imprecise diagnosis and treatment [19]. Multiple studies have identified patterns of accompanying otologic and non-otologic symptoms that help differentiate between otogenic and referred otalgia [19].

#### 4.2.2. Management of TMD-Related Otalgia

Treatment of secondary otalgia related to TMD is focused on resolving the underlying TMJ pathology. Conservative treatment, similar to that outlined in the previous section, is often recommended as the first option for treatment of otalgia related to TMD [49]. Thus, treatment modalities such as therapeutic exercises, occlusal splinting, and massage therapy should be attempted and exhausted prior to progressing to more advanced management. Warm compresses, NSAIDs, and a soft diet help minimize further trauma and tension to the muscles of mastication and TMJ and alleviate symptoms [50]. If otologic pain does not improve with TMD conservative treatment, workup for the abovementioned etiologies of referred otalgia would be warranted, including flexible fiberoptic laryngoscopy and CT/MR imaging.

### 4.3. Tinnitus

#### 4.3.1. Overview and Etiology

Tinnitus, the perception of sound when no external sound is present, is another nonspecific symptom reported in a wide variety of conditions, including otological, neurological, infectious, cardiovascular, metabolic, psychological, medication-induced, and temporomandibular disorder [51]. Presbycusis (age-related hearing loss) remains the most common cause of tinnitus. Damage to inner ear hair cells, whether due to natural deterioration in presbycusis or toxin-mediated, causes auditory processing changes that may present as a ‘buzzing’ or ‘high-pitched’ ringing. However, tinnitus is also notably associated with other otological factors such as loud-noise exposure, trauma, otitis, Meniere’s disease, and vestibular schwannomas [51,52]. Tinnitus can result from neurological conditions such as head injury, whiplash, multiple sclerosis, and vestibular schwannoma (acoustic neuroma), which have been identified as potential contributors. Migraine attacks also commonly cause tinnitus due to disturbances within the central nervous system through vasospasm or trigeminal nerve irritation that may affect blood flow or modulation of sound sensitivity in the auditory cortex. Infections such as otitis media, Lyme disease, meningitis, and syphilis can also play a role in tinnitus onset. Cardiovascular conditions such as hypertension, endocrine and metabolic conditions (e.g., diabetes, hypothyroidism, and hormonal changes), and immune-mediated diseases such as systemic lupus erythematosus and systemic sclerosis have also been implicated in tinnitus development. Psychological factors, particularly anxiety, depression, and emotional trauma, may further exacerbate tinnitus symptoms. Interestingly, tinnitus is also a common side effect of oral medications including salicylates, nonsteroidal anti-inflammatory drugs, aminoglycosides, loop diuretics, and certain chemotherapy agents [51,52].

#### 4.3.2. Classification of Tinnitus

Tinnitus can present with a sudden or gradual onset, can be intermittent or constant, synchronous (pulsatile) or asynchronous (non-pulsatile) with the heartbeat, and of variable intensity. Synchronous or pulsatile tinnitus is less likely to be caused by TMD than asynchronous or non-pulsatile tinnitus, with the former usually associated with benign intracranial hypertension, vascular anomalies, carotid artery or intracranial artery stenosis, or vascular tumors such as glomus tympanicum/jugulare [52].

Another classification of tinnitus is the differentiation between subjective and objective tinnitus. Objective tinnitus can be heard by the examiner and is often caused by venous abnormalities such as aneurysms or myoclonic muscular activity within the middle ear [53]. Subjective tinnitus, which is the TMD form and is also the more common subtype, is the reported perception of sound when no instruments can confirm its existence. Numerous studies suggest that subjective tinnitus may arise from neuroplastic changes in the central auditory system in response to peripheral auditory system damage. This disruption leads to altered neural activity and increased excitability in auditory pathways. As a compensatory mechanism, these neuroplastic changes may contribute to the persistent perception of tinnitus [54,55]. A distinct subtype of subjective tinnitus, known as somatosensory tinnitus, is frequently associated with cervical spine disorders (CSD) and temporomandibular disorders (TMD). Approximately two-thirds of patients with subjective tinnitus experience somatosensory modulation, where head, neck, and jaw movements or muscle contractions can alter the intensity and tone of tinnitus, though the strength of this association remains unclear. Cross-modal interactions in the dorsal cochlear nucleus, where auditory and somatosensory pathways converge, provide a theoretical basis for how TMD may influence tinnitus perception [54,55].

#### 4.3.3. TMD Related Tinnitus

TMD has been cited as a source of both objective and subjective tinnitus [55]. A 2024 systematic review examining both the strength and directionality between TMD and tinnitus found a strong bi-directional association, with tinnitus most prevalent in patients with painful TMD. Notably, 92.9% of tinnitus patients had TMD, compared to 5–31% in the general population, and 57.5% of TMD patients had tinnitus. Tinnitus patients were more likely to have TMD (odds ratio = 2.85) than the reverse (odds ratio = 1.55). Compared to previous systematic reviews, this study found a higher bi-directional prevalence of TMD and tinnitus, potentially due to broader search terms and a larger dataset. The results suggest that tinnitus is more likely a consequence of TMD rather than the reverse [20].

Several mechanisms linking TMD and tinnitus have been proposed; including shared embryological origins, anatomical proximity, neurological pathways, motor innervation, neuromodulation, and stress-related factors; however, there is still no consensus and these mechanisms remain largely theoretical (Table 5). These theories suggest that dysfunction in the temporomandibular joint (TMJ), muscles, and nervous system can directly or indirectly influence auditory perception, contributing to tinnitus. One proposed etiology of tinnitus in the setting of temporomandibular joint disorder suggests that trigeminal nerve irritation in the craniofacial region may play a role. Tinnitus in TMD tends to present clinically similar to the onset and gradual worsening of pain and dysfunction affecting the muscles of mastication; however, some case reports have documented the sudden onset of tinnitus symptoms [51]. Clinical observations suggest that tinnitus in the setting of TMD may occur in a manner similar to aural fullness. The nerves that innervate the jaw muscles and TMJ also contribute to the tone and function of muscles regulating the size of the eustachian tube and the tone of the tympanic membrane. Dysfunction in these neuromuscular pathways can potentially contribute to tinnitus symptoms. Another possible mechanism involves the sphenomandibular ligament (SML), also referred to as the malleomandibular ligament (MML) or anterior ligament of the malleus (AML), which connects the malleus in the middle ear to the mandible. This ligament is a fibrous remnant of Meckel’s cartilage, an embryonic structure that contributes to the development of the lower jaw. In cases of temporomandibular joint dysfunction, slight changes in mandibular position can create ligamentous traction on the malleus, altering its function and contributing to tinnitus [56]. Recognizing these interconnected pathways is essential for multidisciplinary approaches in both diagnosis and treatment.

#### 4.3.4. Treatment Approaches and Efficacy

Several studies have explored the effectiveness of TMD-targeted therapies in alleviating tinnitus symptoms; however, the strength of their effect on tinnitus remains unclear. A systematic review aimed to assess whether TMD treatment positively affects tinnitus symptoms, found that conservative TMD treatments, particularly a combination of splint therapy and exercise, are the best investigated treatment approach, showing a reduction in tinnitus severity and intensity. Despite promising findings, limitations persist, including inconsistent diagnostic criteria, a lack of standardized tinnitus outcome measures, and the absence of control groups, all of which reduce statistical reliability. Additionally, psychosocial factors such as stress, anxiety, and depression are often overlooked, despite their role in both TMD and tinnitus severity [21].

Individual studies further support the potential benefits of TMD therapy on tinnitus symptoms. Mahmoudian et al. (2023) [30] examined six male patients with chronic non-pulsatile tinnitus receiving TMD therapy and found significant improvements in tinnitus intensity, annoyance, and awareness, though audiometric thresholds remained unchanged. These findings suggest that TMD therapy may alleviate tinnitus in patients with somatosensory tinnitus and concurrent TMD. A randomized controlled trial from the journal Pain Medicine conducted by Delgado de la Serna et al. proposed at-home exercises and education in combination with cervico-mandibular manual therapy performed by an experienced physical therapist. This therapy involves mandible distraction intervention and soft tissue mobilization of the masseter, temporalis, sternocleidomastoid, and upper trapezius muscles. Participants receiving cervico-mandibular manual therapy showed significantly improved tinnitus symptoms when compared to those receiving education and at-home therapy alone [22]. The results suggest that a comprehensive therapeutic approach is beneficial. However, further research is needed to confirm intervention effectiveness.

Overall, the precise pathophysiology of tinnitus in patients with TMD remains poorly understood, although conservative management techniques have been shown to improve symptoms regardless [58].

### 4.4. Vertigo

#### 4.4.1. Overview and Etiology

The vestibular system within the inner ear is responsible for maintaining the sensation of balance and orientation in space. Dysfunction of this system may lead to vertigo, a sensation of constant motion and dizziness. Vertigo is a symptom of many conditions and may have central or peripheral causes. Peripherally, within the otologic realm, the most common diagnoses of vertigo include benign paroxysmal positional vertigo (BPPV), vestibular migraines, Meniere’s disease, vestibular neuritis, and labyrinthitis. Although some studies have reported statistically significant associations between temporomandibular joint disorder (TMD) and vertigo, the underlying pathophysiology remains unclear [23].

**Figure 3:**
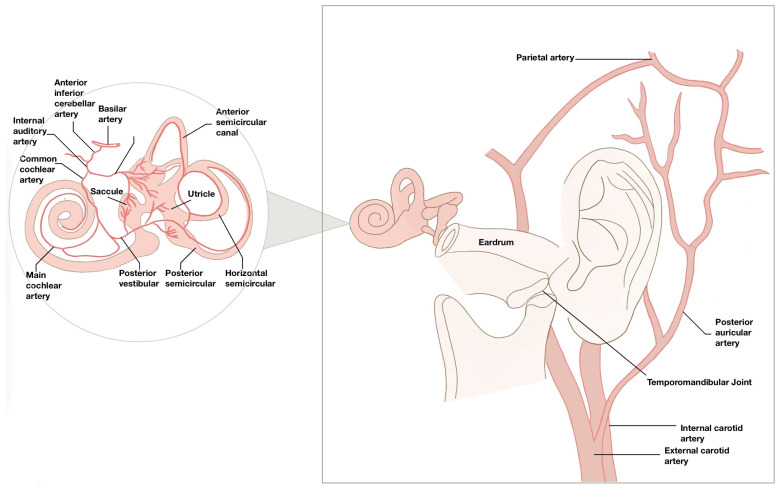
Proposed mechanisms linking temporomandibular disorders and vertigo. Schematic illustration demonstrating (**left**) the inner ear vestibular apparatus and vascular supply and (**right**) the anatomic proximity of the temporomandibular joint to adjacent neurovascular structures, including branches of the carotid system, which have been proposed as potential contributors to vertigo symptoms in temporomandibular disorders.

Williamson proposed two possible mechanisms to explain vertigo in the context of temporomandibular disorders. The first suggests that noxious stimuli within the peri-discal tissues may trigger a sympathetic reflex via the carotid plexus, leading to vasoconstriction of the internal auditory and posterior auricular arteries and thereby reducing blood flow to the inner ear. Additionally, pressure on the foramina connecting the glenoid fossa to the middle ear cavity, where the vascular plexus communicates, may also reduce blood flow. The second hypothesis, introduced by Myrhaug, posits that spastic contraction of the stapedius muscle may cause abrupt movement of the stapes, disturbing peri-lymphatic fluid in the inner ear and triggering dizziness (Figure 3) [59]. However, a direct anatomical or physiological connection between TMD and vertigo has not been conclusively established. The frequent co-occurrence of TMD and BPPV may be attributable to their high individual prevalence rather than a direct anatomical or physiological connection.

#### 4.4.2. TMD Related Vertigo

Several studies have explored the association between TMD and vertigo. Chole and Parker (1992) [23] conducted a retrospective study to assess the prevalence of vertigo and tinnitus in patients with TMD (*n* = 338) compared with age-matched controls (*n* = 694). The study found that tinnitus and vertigo symptoms were significantly more prevalent in the TMD group compared to the control groups [23]. Aldè et al. performed a retrospective analysis examining otologic symptoms in individuals with TMD and reported that 19.8% of patients experienced new-onset vertigo [24]. Additionally, Marchiori et al. (2014) [25] conducted a cross-sectional study focusing on elderly individuals (mean age: 69.23  ±  5.70 years), comprising 127 women and 73 men. The study included 199 participants, of whom 141 had clinically diagnosed TMD, and 58 did not. Among those with TMD, 54 individuals (27.14%) reported vertigo, compared to only 12 individuals (6.03%) in the non-TMD group, further supporting the possible association between TMD and vestibular symptoms [25]. While these studies suggest a possible correlation between TMD and vertigo, they are limited by small sample sizes, reliance on self-reported symptoms, and study designs that do not allow for causal inference.

#### 4.4.3. Treatment Approaches and Efficacy

Oral splint therapy has been shown to improve symptoms in patients suffering from TMD in combination with vertigo. A long-term controlled study by Bernkopf et al. (2022) demonstrated that oral splint therapy led to sustained improvements in vertigo symptoms in patients with both TMD and Meniere’s disease, with effects lasting over two years [26].

### 4.5. Hearing Loss

#### 4.5.1. Etiology and Classification

Hearing loss is categorized as either conductive, sensorineural, or a combination of both. Conductive hearing loss (CHL) occurs when a physical blockage in the conductive system of the ear prevents the mechanical transmission of sound waves. Typical etiologies of CHL include foreign body in the ear, cerumen impaction, tympanic membrane perforation, ossicular chain disarticulation, or middle ear masses. Sensorineural hearing loss (SNHL) occurs when acoustic vibrations are transmitted to the inner ear but cannot be converted into neural impulses in the cochlea. The etiology of SNHL is diverse and often multifactorial, broadly categorized as congenital or acquired. Congenital SNHL may be due to genetic syndromes like Pendred or Usher or caused by perinatal factors such as birth asphyxia, jaundice, or ototoxic exposure. Acquired SNHL is more common and includes a wide range of etiologies such as viral and bacterial infections, prolonged noise exposure, ototoxic drug use, age-related presbycusis, trauma, systemic diseases (e.g., diabetes, hypertension), autoimmune conditions, and vestibular schwannomas [60]. The proposed pathophysiology linking hearing loss and TMD includes dysfunction or tonic spasm of the tensor tympani muscle, as well as damage to the malleus in the ossicular chain due to its connection to the temporomandibular complex via the disco-malleolar ligament. As this anatomical mechanism breaks down, the transmitting system causes middle ear dysfunction, resulting in conductive hearing loss [38].

#### 4.5.2. TMD Related Hearing Loss

The relationship between hearing loss and temporomandibular disorders (TMD) remains inconclusive, with some studies reporting correlations but lacking clear causality. Baldursson and Blackmer (1987) [27] observed a notable correlation between midfrequency sensorineural hearing loss and TMD symptoms, though they found no evidence of a direct causal relationship. The authors proposed that treatments involving extensive manipulation of oral structures, particularly in dentistry, orthodontics, and oral surgery, may lead to acoustic trauma via bone conduction, potentially damaging inner ear structures and causing hearing deficit [27]. Similarly, other studies note mild sensorineural hearing loss and type B and C tympanograms to be significantly associated with TMD compared to controls [28]. In contrast, a controlled study by Toller and Juniper (1993) comparing 57 patients with temporomandibular joint (TMJ) dysfunction to 57 age- and gender-matched controls found no statistically significant changes in audiometric findings in patients with TMD [29].

## 5. Conclusions

In summary, the literature suggests an association between diverse otologic symptoms and TMD, but there is little consensus on the best management for these symptoms. However, interpretation of prevalence estimates should be undertaken with caution because substantial heterogeneity exists across the available literature. Included studies differed with respect to patient populations, study design, diagnostic criteria for TMD, definitions of otologic symptoms, and methods of symptom assessment. Consequently, reported prevalence values may not be directly comparable across investigations and should be interpreted as representative ranges rather than uniform estimates. Furthermore, the available evidence encompasses a broad range of study designs, including systematic reviews, meta-analyses, randomized controlled trials, observational studies, case series, and case reports. As a result, the strength of evidence varies considerably among reported findings, and conclusions supported by higher-level evidence should be interpreted with greater confidence than observations derived primarily from small case series or individual case reports. Additionally, this review was based exclusively on the PubMed database; therefore, eligible publications indexed in other databases may not have been captured.

Many case studies have proposed conservative TMD management to provide symptomatic relief, but the exact mechanism of this connection remains unproven. Reported rates of symptom improvement should also be interpreted cautiously, as many studies were observational in nature and lacked control groups. Furthermore, differences in treatment protocols, outcome measures, follow-up duration, and patient populations may contribute to variability in reported treatment outcomes across studies. Traditional TMD management techniques are generally safe and preliminary evidence suggests efficacy, although additional studies are warranted to confirm their effectiveness in patients presenting with these otologic symptoms.

## Figures and Tables

**Table 1:** Summary of included studies reporting otologic symptoms associated with temporomandibular disorder (TMD) [2,13,14,15,16,17,18,19,20,21,22,23,24,25,26,27,28,29,30].

Author(s), Year	Title/Study Focus	Methodology	Sample/Context	Key Findings
Porto de Toledo et al., 2017 [2]	Prevalence of otologic signs and symptoms in adult TMD patients	Systematic review and random-effects meta-analysis of 8 observational studies	Total aggregated adult sample size *n* = 1040	Aural fullness: 74.8% (SD 43.0–96.3%); Otalgia: 55.1% (SD 31.8–77.3%); Tinnitus: 52.1% (SD 38.4–65.7%); Vertigo: 40.8% (SD 11.3–74.7%); Hearing loss: 38.9% (SD 2.8–85.5%).
Chole RA, Parker WS, 1992 [23]	Tinnitus and vertigo in patients with temporomandibular disorder	Cross-sectional, case–control survey	338 patients with TMD vs. 694 age- and sex-matched controls (dental clinic and general population)	Tinnitus reported by 59% of TMD patients vs. 12% of dental controls and 6% of general population controls. Vertigo reported by 40% of TMD patients vs. 9% of dental controls and 5% of general controls (*p* < 0.001).
Aldè M et al., 2022 [24]	Prevalence of new onset otologic symptoms in patients with temporomandibular disorders	Retrospective observational study	400 patients with TMD (301 females, 99 males); median age 39.6 ± 15.6 years	76% of TMD patients reported ≥ 1 otologic symptom. Aural fullness was most common (33.3%), followed by tinnitus (23%) and vertigo (19.8%).
Marchiori LL et al., 2014 [25]	Probable correlation between temporomandibular dysfunction and otologic symptoms	Cross-sectional observational study	776 patient charts reviewed (344 with TMD vs. 432 without TMD)	Otologic symptoms reported by 59.9% of TMD patients vs. 29.2% of non-TMD controls. Tinnitus, vertigo, otalgia, and hearing loss were significantly more prevalent in the TMD group.
Baldursson G, Blackmer ER, 1987 [27]	Temporomandibular joint symptoms in patients with midfrequency sensorineural hearing loss	Cross-sectional case–control study	50 adults with 1–2 kHz audiometric “notch” vs. 50 matched controls	TMJ symptoms (pain, tenderness, joint noises, bruxism/clenching) were significantly more frequent in the hearing loss group.
De La Torre Canales G et al., 2024 [20]	Associations between temporomandibular disorders and tinnitus	Systematic review and meta-analysis	32 observational studies from electronic databases	Among TMD patients, 57.5% had tinnitus; among tinnitus patients, 92.9% had TMD. Strong bidirectional association (OR for TMD → tinnitus = 1.56; OR for tinnitus → TMD = 2.86; *p* < 0.001).
Peng Y, 2017 [14]	Temporomandibular joint disorders as a cause of aural fullness	Retrospective clinical case series	112 patients from Beijing ENT clinic with aural fullness as primary complaint	All patients had aural fullness without detectable otologic disease. Symptoms resolved completely in 67 patients and significantly improved in 34 (90.2% overall effectiveness), especially in muscle-related TMD.
de Felício CM et al., 2008 [16]	Otologic symptoms of temporomandibular disorder and effect of orofacial myofunctional therapy	Randomized controlled clinical trial	20 patients with articular TMD and 8 asymptomatic controls	Baseline symptoms included ear fullness (90%), otalgia (65%), tinnitus (60%). Significant reductions in ear fullness, otalgia, and tinnitus after therapy, with improved muscle coordination.
Delgado de la Serna P et al., 2020 [22]	Effects of cervicomandibular manual therapy in patients with temporomandibular pain disorders and somatic tinnitus	Randomized clinical trial	61 adults with somatic tinnitus attributed to TMD	Manual therapy group showed significantly greater reductions in tinnitus severity and THI scores, along with improvements in TMD pain, pressure pain thresholds, and mandibular range of motion.
Bernkopf E et al., 2022 [26]	Oral splint therapy in patients with Ménière’s disease and temporomandibular disorder	Retrospective case–control study	63 patients with Ménière’s disease and TMD (37 treated with splints, 26 untreated)	Vertigo control achieved in 86.5% of treated patients vs. 19.2% of controls. Hearing loss and tinnitus severity improved; no significant change in aural fullness.
Hernández-Nuño de la Rosa MF et al., 2022 [13]	Is there an association between otologic symptoms and temporomandibular disorders?	Evidence-based review	Peer-reviewed studies covering otologic complaints in TMD (multiple designs)	No consensus on optimal management. Conservative TMD therapies may reduce aural fullness, otalgia, tinnitus, hearing loss, and vertigo; interdisciplinary care recommended.
Stechman-Neto J et al., 2016 [17]	Effect of conservative temporomandibular disorder therapy on otologic signs and symptoms	Systematic review	8 clinical and observational studies evaluating conservative TMD therapies	Most studies reported partial or complete improvement of otologic symptoms after therapy; evidence insufficient to definitively support or refute treatment effectiveness.
Naderi Y et al., 2023 [18]	Temporomandibular treatments are significantly efficient in improving otologic symptoms	Prospective observational cohort with interventional therapy	40 patients with TMD and unexplained otologic symptoms	>50% reported partial or complete symptom recovery (*p* < 0.05). Otalgia most common (95%), followed by tinnitus (42.5%), ear fullness (30%), and dizziness (27.5%).
Michiels S et al., 2019 [21]	Does conservative TMD therapy affect tinnitus complaints?	Systematic review of clinical and cohort studies	11 studies of splint and exercise therapies	Most studies showed reductions in tinnitus severity and intensity; overall evidence quality low due to heterogeneity and methodological limitations.
Mahmoudian S et al., 2023 [30]	Conservative TMD treatment effect on tinnitus	Case series	6 patients with chronic non-pulsatile tinnitus and concomitant TMD	Significant improvements in tinnitus severity, intensity, and awareness (VAS; *p* < 0.05). TMD disability scores also significantly decreased (*p* = 0.0001).
Effat KG, 2016 [28]	Otological symptoms and audiometric findings in temporomandibular disorder patients	Prospective case–control observational study	104 TMD patients vs. 110 controls evaluated with pure-tone audiometry	High frequency of otologic symptoms reported. 25% of TMD patients had mild sensorineural hearing loss compared with controls (*p* = 0.001).
Toller MO, Juniper RP, 1993 [29]	Audiological evaluation of aural symptoms in temporomandibular joint dysfunction	Case–control audiological assessment	57 TMD patients vs. 57 age-matched controls	No significant differences in audiometry, tympanometry, or Eustachian tube tests. Minor increase in middle-ear compliance in female TMD patients only.
Dalla-Bona D et al., 2015 [15]	Unilateral ear fullness and temporary hearing loss managed as TMD	Single-patient case report	A female with persistent ear fullness after failed ENT interventions	~90% resolution of ear fullness and hearing loss after conservative TMD therapy including steroid injections.
Kim SH et al., 2015 [19]	Clinical differences in types of otalgia	Cross-sectional observational study	294 patients presenting with otalgia at ENT clinics	29.3% had referred otalgia; TMD was a prominent cause. Referred otalgia was more common in women and adults.

Abbreviation: ENT, otolaryngology; OR, odds ratio; PPTs, pressure pain thresholds; ROM, range of motion; THI, Tinnitus Handicap Inventory; TMD, temporomandibular disorder; TMJ, temporomandibular joint.

**Table 2:** Effectiveness of conservative temporomandibular disorder (TMD) therapy in patients presenting with aural fullness (*n* = 112) [14].

TMD Classification Group	No. of Patients	No. Improved/Resolved, *n* (%)
Muscle disorders	68	64 (94.1) *
Disc displacements	39	33 (84.6)
Arthralgia/osteoarthritis/osteoarthrosis	5	4 (80.0)

* Of 68 patients with muscle disorders, 58 improved following physiotherapy. Abbreviation: TMD, temporomandibular disorder.

**Table 3:** Structures and mechanisms associated with otalgia in temporomandibular disorders.

Structure/Pathway	Proposed Mechanism	Key Studies/Findings
Auriculotemporal nerve	Supplies the anterior/superior walls of the external auditory meatus and tympanic membrane	Tension or irritation in this nerve is linked to ear pain.
Sphenomandibular ligament [38]	Excessive tension may damage the middle ear via the anterior malleolar ligament	Hypothesized contributor to otologic symptoms
Deep masseter and medial pterygoid muscles [31]	Referred pain from myofascial trigger points in these muscles may radiate to the ears	Simons et al.
Posterior belly of digastric [31]	Trigger points may contribute to referred ear pain	Simons et al.
Lateral pterygoid muscle [39,40]	May refer pain to the ear due to anatomic/functional proximity	Fricton et al., Wright.
Temporalis tendon [41]	Tendinitis may refer pain to the ear, particularly near insertion at the coronoid process	Dupont and Brown

Abbreviation: TMD, temporomandibular disorder.

**Table 4:** Symptom patterns associated with otogenic versus referred otalgia [19,42].

Symptoms	Otalgia Type *	Supporting Studies
Ear fullness, hearing loss, otorrhea, tinnitus, dizziness	Otogenic	[19,42]
Rhinorrhea, nasal obstruction, postnasal drip, sore throat, fever, voice change, reflux symptoms	Referred	[19,42]

* Otogenic otalgia originates from pathology within the ear, whereas referred otalgia originates from extra-auricular sources.

**Table 5:** Proposed theories linking temporomandibular disorders (TMD) and tinnitus [57].

Theory	Description
Embryological Development	The medial pterygoid, tensor veli palatini, and tensor tympani muscles originate from the first pharyngeal arch, explaining their structural and functional link to both jaw and ear function.
Anatomical Proximity	The TMJ and middle ear are closely positioned. Dysfunction in the mandibular condyle can compress nerves and ligaments (e.g., auriculotemporal nerve, disco-malleolar ligament), leading to tensor tympani muscle contraction and tinnitus.
Neurological Overlap	The trigeminal (V), facial (VII), glossopharyngeal (IX), vagus (X) nerves, and cervical plexus (C2, C3) share pathways connecting jaw function with ear sensitivity. Dysfunction in these nerves may alter pain perception and muscle reflexes, contributing to tinnitus.
Motor Innervation	The trigeminal nerve (mandibular branch) controls both masticatory muscles and the tensor tympani muscle. Excessive tension in one group can lead to dysfunction in the other, potentially causing tinnitus.
Neuromodulation	Sensory stimuli from the head and neck interact with auditory processing centers. Cross-modal plasticity may cause abnormal sensory interactions, contributing to tinnitus development.
Stress	Chronic stress is linked to both TMD and tinnitus, increasing muscle excitability, pain perception, and neurotransmitter imbalances (serotonin, catecholamines). Stress management has been shown to reduce both TMD pain and tinnitus severity.

Abbreviations: TMD, temporomandibular disorder; TMJ, temporomandibular joint.

## Data Availability

No new data were created or analyzed in this study. Data sharing is not applicable to this article.
